# A Camera Sensors-Based System to Study Drug Effects on In Vitro Motility: The Case of PC-3 Prostate Cancer Cells

**DOI:** 10.3390/s20051531

**Published:** 2020-03-10

**Authors:** Maria Colomba Comes, Arianna Mencattini, Davide Di Giuseppe, Joanna Filippi, Michele D’Orazio, Paola Casti, Francesca Corsi, Lina Ghibelli, Corrado Di Natale, Eugenio Martinelli

**Affiliations:** 1 Dept. Electronic Engineering, University of Rome Tor Vergata, 00133 Roma, Italy; maria.colomba.comes@uniroma2.it (M.C.C.); di.giuseppe@ing.uniroma2.it (D.D.G.); filippi@ing.uniroma2.it (J.F.); michele.dorazio10@gmail.com (M.D.); casti@ing.uniroma2.it (P.C.); dinatale@uniroma2.it (C.D.N.); martinelli@ing.uniroma2.it (E.M.); 2 Dept. of Chemical Science and Technologies, University of Rome Tor Vergata, 00133 Roma, Italy; francesca.corsi@uniroma2.it; 3 Dept. Biology, University of Rome Tor Vergata, 00133 Roma, Italy; ghibelli@uniroma2.it

**Keywords:** camera sensor, cell-motility, drug effect on in-vitro, prostate cancer cells

## Abstract

Cell motility is the brilliant result of cell status and its interaction with close environments. Its detection is now possible, thanks to the synergy of high-resolution camera sensors, time-lapse microscopy devices, and dedicated software tools for video and data analysis. In this scenario, we formulated a novel paradigm in which we considered the individual cells as a sort of sensitive element of a sensor, which exploits the camera as a transducer returning the movement of the cell as an output signal. In this way, cell movement allows us to retrieve information about the chemical composition of the close environment. To optimally exploit this information, in this work, we introduce a new setting, in which a cell trajectory is divided into sub-tracks, each one characterized by a specific motion kind. Hence, we considered all the sub-tracks of the single-cell trajectory as the signals of a virtual array of cell motility-based sensors. The kinematics of each sub-track is quantified and used for a classification task. To investigate the potential of the proposed approach, we have compared the achieved performances with those obtained by using a single-trajectory paradigm with the scope to evaluate the chemotherapy treatment effects on prostate cancer cells. Novel pattern recognition algorithms have been applied to the descriptors extracted at a sub-track level by implementing features, as well as samples selection (a good teacher learning approach) for model construction. The experimental results have put in evidence that the performances are higher when a further cluster majority role has been considered, by emulating a sort of sensor fusion procedure. All of these results highlighted the high strength of the proposed approach, and straightforwardly prefigure its use in lab-on-chip or organ-on-chip applications, where the cell motility analysis can be massively applied using time-lapse microscopy images.

## 1. Introduction 

The advent of sophisticated camera sensors integrated into time-lapse microscopy (TLM) devices coupled with modern software tools for video and data analysis, allowed increasing capabilities to “see in the deep” [1]. 

Thanks to these facilities, the cellular microenvironment and related biological mechanisms can be now investigated online during the phenomena evolution, letting research to understand the dynamics of the processes involved for a deeper understanding of the biological mechanism. The online analysis allowed by such sensors permits us to follow and analyze cellular movement in the environment, and to further extract quantitative kinematics descriptors from the cell trajectories, therefore giving to cell motility a crucial role. So, it is possible to change the measurement paradigm going from the macro-temporal scale (acquisition every many hours for a static investigation), to a temporal scale of the order of minutes. Virtually, the individual cells can be considered themselves as a sort of sensitive element of a sensor that uses the camera as a transducer, and provides the movement of the cell as an output signal that is able to retrieve information about the chemical composition of the close environment. 

Cell-based sensors have been largely used in cellular physiological parameter detection, human olfactory mimicking systems [2,3,4,5,6], the treatment effect analysis, an environmental toxicity test, and immunotherapy efficacy [7,8,9]. 

Thanks to the incremented uptake provided by time-lapse microscopy (TLM) and computer simulations for data analysis, it is possible to simultaneously analyze the motion of many cells, and to track their morphological as well as motion changes during cell life. This is possible now also, thanks to the increased spatial and temporal resolution of the camera sensors and related electronic interfaces [10]. Such aspects are going to be ever more relevant, since, as well known, they can be related to alterations in the chemical compounds in the culture (drug administering), cell signaling (cell–cell interaction), DNA damage, cell metabolism, etc. In addition, the coordinated motility of group of cells (e.g., cancer cells clustered) may produce unusual behavior for individual cells, such as emergent chemotaxis [11] or cell leader/follower paradigmatic roles in movement coordination [12]. 

According to the fact that cell movement depends upon the neighboring environment, the aim of this study is to exploit cell movement as the output signal of a “virtual sensor”, whose sensitive element is the cell itself. The observed output signal is then the cell trajectory extrapolated from the time-lapse microscopy images. 

Even if cell tracks have been extensively studied in many fields, from the morphogenesis of pluricellular organisms [13] to adult physiological processes (such as tissue repair and immune cell trafficking) [14], and many cancer-related diseases (such as cancer metastasis) [15,16], it is not straightforward to construct a unique motion model for the entire cell track. 

Experimental evidences, after all, have already suggested that the single cell track can be divided in different parts each one characterized by a specific motion kind [17,18,19,20,21,22,23]. Starting from the hypothesis that each single motion kind carried a different representation of the information of the cell, and of its interaction with the environment [19,20] (e.g., anomalous diffusion may be a method for cells to localize membrane receptors and control intramembrane signaling), the most innovative aspect in this work is that we considered each single sub-track (and not simply the entire trajectory) as a sort of sensor (Cell Motion Sensor, CMS). In such a way, each cell track can be virtually represented by an array of CMSs, a “Sensor Array”, within which each single sub-track can be characterized by a specific motion kind. The number of identified CMSs may change from cell track to cell track, but CMSs can be coupled to three possible well-known typologies of motion: *isotropic random walk*, *random walk with drift* and *confined/sub-diffusive random walk* [18] (a sort of three different input–output curves).

In the last past decades, diverse attempts have been presented with the aim of identifying these different modes of motions [18,19,20,21,22,23,24,25]. According to the state-of-the-art scenario, we decided to use here the Moment Scaling Spectra (MSS) approach that was demonstrated to be more effective in determining different motion modes along a track. 

The information of each single sub-track can be then extracted through the kinematics descriptors of the trajectory. These features then represent the input to a distinct classification model for each kind of motion. 

As a proof of concept, we here applied the proposed system to the study of the effects of a chemotherapeutic drug (topoisomerase II inhibitor etoposide) at different concentrations on prostate cancer cells PC-3 cultured in a 35 mm Petri dish, and spontaneously grouped into clusters [26]. It will be demonstrated that the division in sub-tracks allows for improving the recognition performance of the drug effect on the cell motility patterns (i.e., paradigm “sensor array”), with respect to the analysis of the kinematics descriptors computed on the entire cell track (i.e., paradigm “single cell-based sensor”); furthermore, we will also prove that the analysis of classification results mediated over the descriptors extracted at cluster levels can be the optimal solution (i.e., in a new paradigm of “sensor fusion” strategy).

The present study overcomes the criticisms and the limitations highlighted in the preliminary work by Di Giuseppe et al. [26] that was aimed at verifying the effectiveness of cell motility to discover block replication effects on cancer cells. In particular, we present here a novel way to analyze the information content of cell trajectories, considering the track as the concatenation of separate signals that come from a different motion kind. With this approach, each cell trajectory can be seen as the signal of an array of cell-based sensors constructed upon the different sub-tracks of a single cell track. Numerical results obtained in the classification of different cell tracks under different drug concentrations incredibly improve the results achieved by the old algorithms, either in terms of the number of cells to analyze, the automatic way to operate, as well as the variety of drug concentrations tested. Furthermore, the more general approach presented here will translate in a fully automatic way the concept of selecting the best training examples for constructing the recognition model, leading to the so-called “good teacher selection” strategy. This new approach focuses on the crucial selection of the best samples for the model construction beyond the standard selection of the best features. 

The larger number of experiments with additional biological conditions (drug concentrations), and the increased number of videos considered here will further demonstrate the reliability of the proposed strategy, totally encompassing the single-cell sensor concept towards a model that better represents, not only the complexity of a group of cells with coordinated actions, but even the strong complexity underhand of the life of each single cell. 

Finally, the present algorithm proposes a twofold change of perspective in cell motility investigation. In the first place, the novel association cell track-sensor array plays a key role in the better discrimination of the drug effect respect to Di Giuseppe et al. [26]. Secondly, the division of cell trajectories in sub-tracks is not restricted to consider cells on its own as in Montiel et al. [22] or Dosset et al. [23]. Indeed, the approach is based on the identification of multiple diffusive motion kinds according to the interaction forces among closer cells. The influence of cells on each other also results in a collective response of cells belonging to the same cluster as the drug [11,12,13].

## 2. Materials and Methods

### 2.1. A Sketch of the Method

**Figure 1:**
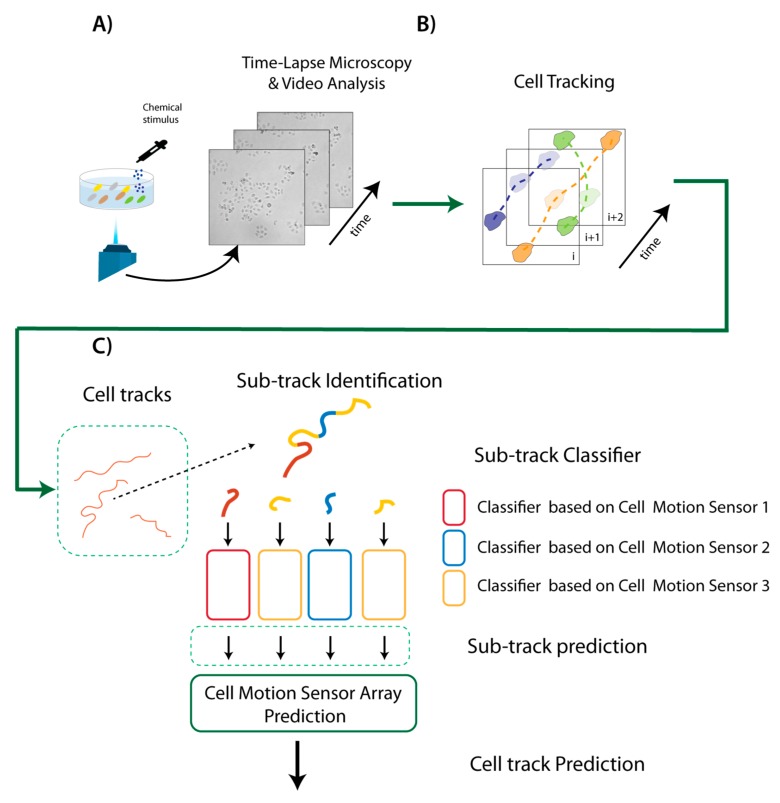
A Schematic representation of the whole sensor platform. (**A**) The living cells sample is prepared and put into a Petri dish. (**B**) Time-lapse Microscopy analysis is used to acquire the video of moving cells. Cells are located, differentiated and individually tracked. (**C**) Each trajectory is segmented into different sub-tracks, each representing a different Cell Motion Sensor (CMS). By combining the responses of more CMSs using majority voting, an estimation of drug effects is provided.

The whole approach is summarized in Figure 1. First, a live cell sample is prepared and put into a Petri dish. Then, time-lapse microscopy (TLM), coupled with video analysis, is used to acquire and analyze the video sequence of cells under the microscope. First, unsupervised cell clustering is applied by automatically assigning each cell to a specific cluster in the video sequence. Cells are then localized and tracked by *Cell-Hunter* software [26,27,28], in order to automatically identify a set of individual cell trajectories within the video. Each trajectory is further segmented into different sub-tracks, each representing a different Cell Motion Sensor (CMS). Three different motion-based classifiers are constructed according to the previous discussed model of motions, and applied to the corresponding sub-track, providing a sub-track prediction result. 

A unique classification result is then achieved by using majority voting, i.e., we combined the responses provided by more CMSs (i.e., by different sub-tracks in the same cell track) in the array represented by the entire cell track. The final assigned label quantifies the cancer treatment effect on each specific cell. 

### 2.2. Cell Culture

PC-3 human prostate cancer cells, cell line initiated from a bone metastasis of a grade IV prostatic adenocarcinoma, were grown at 37 °C in a 5% CO_2_ humidified atmosphere in Roswell Park Memorial Institute (RPMI) 1640 medium, supplemented with 10% fetal bovine serum (FBS), 2 mg/mL glutamine, 100 IU/mL penicillin and streptomycin (Euroclone). We applied the CMS device to the study of the motility of PC-3 cells cultured in a 35 mm Petri dish environment grown to 40–60% confluence. Cells were treated with the topoisomerase II inhibitor etoposide (chemotherapeutic drug, Sigma) at the final concentrations of 0.0 μM, 0.5 μM, 1.0 μM, 5.0 μM and 50 μM, and immediately analyzed by time-lapse microscopy for the first 6 h. 

### 2.3. Experimental Set-Up

Time-lapse videos have been acquired with a customized small-scale inverted microscope for live-cell imaging. The experiments have been recorded in brightfield (8-bit grayscale) with a 10X magnification, capturing one frame every minute, with 6 h of total video time. The samples were exposed to light only for the acquisition (5–7 s per frame), in order to prevent any photo-activated cell mechanisms. The resulting videos have a FOV of 1.2 mm width by 1 mm height with a theoretical spatial resolution of 0.33 µm/pixel.

### 2.4. Video Analysis

In the following, we present the three main steps of the video data analysis: automatic clustering and tracking, sub-track identification within a trajectory, and feature extraction.

#### 2.4.1. Automatic Clustering and Tracking

**Figure 2:**
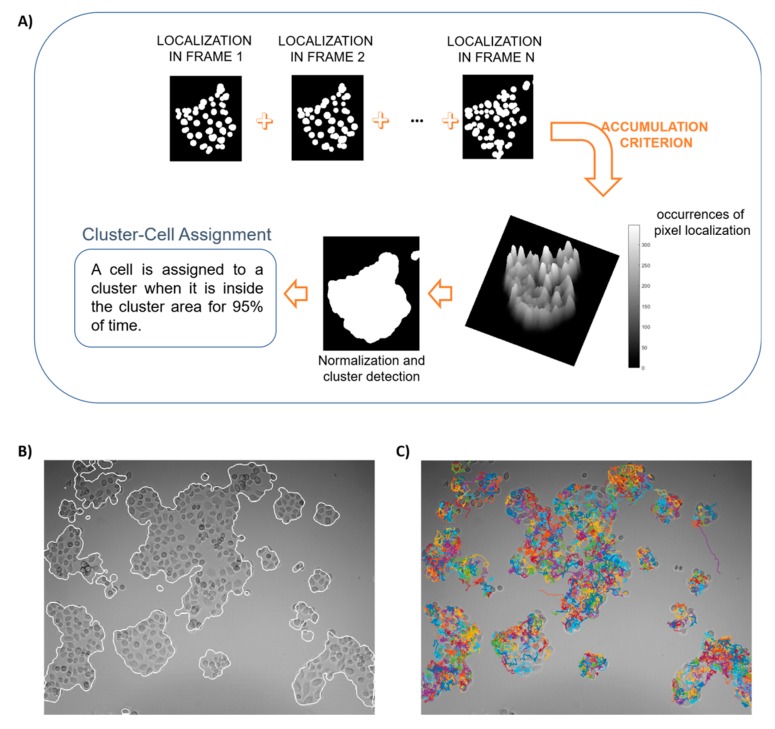
A schematic representation of automatic cell clustering and tracking. (**A**) Automatic cluster-cell assignment procedure. (**B**) Visual result of the automatic cell clustering. (**C**) Cell tracking results superimposed on the first video frame.

An unsupervised clustering technique (Figure 2A) is applied to detect clusters in the videos of our experimental scenarios. The technique is based upon the localization of individual cells through the segmentation of circular-shaped objects using the Circular Hough Transform (CHT) [29], with its radius settled around the mean manually-estimated radius of the considered cells. 

At each frame, after cell detection, each cell nucleus is represented by a white circular object. In order to obtain the entire cluster segmentation, an accumulation criterion, consisting of the overlapping of the cell nuclei detected along all of the frames, was applied. In Figure 2B, an example of the final cluster detection result is shown. A grayscale map is first obtained, where a higher intensity indicates cells with limited motility, and hence higher probability to stay in that position during movement. Lower intensities indicate minor probability for cells to be located in that position. Pixel intensity thresholding by the Otsu approach [30] is then applied to recover a binary (black and white) image, where white objects indicate a meaningful cell localization probability. The contouring of detected regions define cluster boundaries. 

As a second step, we applied the software *Cell Hunter* [26,27,28] for Single Particle Tracking (SPT) [31] to identify cell trajectories in the video. An example of this application is represented in Figure 2C. 

The last stage relies upon a univocal cluster-cell assignment. The definitive cell allocation was preceded by a time-varying procedure, which assigned each cell position at a time to a cluster by computing the time-by-time Euclidean distance between the cell center in each frame and the cluster region center. 

Most cells remained on the same cluster for the entire duration of the experiment, a few others migrated from one cluster to another. We assigned such cells to the main cluster, i.e., the cluster in which cell trajectories fell for most of the time, when they were inside this cluster for 95% of the time.

Clusters with less than 10 cells have been excluded from the analysis, so that each cluster could be considered as a sufficiently large independent population to measure the mutual influence among cells, and the collective response to the drug (see the sub-paragraphs below).

#### 2.4.2. Sub-Track Identification within a Trajectory

Several approaches have been proposed in the state-of-the-art to implement the sub-track motion estimation [22,23]. In the work of Montiel et al. [22], the authors detected changes in diffusion modes, i.e., confined, isotropic and directed random walks, within one cell trajectory, by referring to a statistical approach, a based likelihood ratio test, and by deriving Maximum Likelihood Estimators to estimate diffusion coefficients related to the diverse identified modes.

Dosset et al. [23], instead, trained a back-propagation neural network (BPNN) to distinguish the three diffusion behaviors within a single cell trajectory, then classified by means the computation of the Mean Squared Displacement [18].

The aforementioned methodologies were applied to individual trajectories by disregarding the mutual influences of neighboring cells. However, according to recent works [32,33,34,35], the interplay among cells and their motion behavior in concert have characterized the core of cell motility items. Mathematical models have been designed to describe coordinated cell migration [32,33,34,35]. They reserved a valuable role in cell–cell interactions to explain the coordinated cell motion. On this basis, to design our approach of diffusion modes identification, we assumed that the transition between motion kinds within the same cell trajectory in a cluster was strongly influenced by the presence of the other cells of the same cluster and by the consequent interaction forces among them. 

After computing the interaction force acting on cells separately, we considered some local minima of the force (explained later) as the motility transition between a motion mode to another, because they correspond to a lack of interaction (i.e., as a breakup in the single cell motility) within the cluster.

For the definition of the interaction force, we readapted the concept of interaction among cells in wound healing [35] to cells inside the same cluster.

Let c represent an arbitrary cell belonging to a cluster C. The interaction force exerted onto the considered cell c by the other cells of the same cluster C was computed as the sum of the gradients of attractive Gaussian potentials, given by

(1) $$\vec{F^{c}}=\left(F_{x}^{c},F_{y}^{c}\right)=-\underset{k\in C}{\sum }\nabla U_{k}^{c}\left(x,y\right)$$

where

(2) Ukc=−U0exp(−(rkctol)2)              

expresses the Gaussian potential related to the $k^{th}$ cell of the cluster with amplitude $U_{0},$ chosen as $U_{0}=1\;\mu m^{2}/min$. Because of this assumption, the force was called a normalized interaction force. 

Since the center of the attraction is the considered cell c, $r_{k}^{c}$ denotes the distance between the cell c and the $k^{th}$ cell of C, whilst tol is the attractive potential range, the maximum cancer cell–cell overlapping distance, imposed equals to the mean-diameter of detected cells. 

**Figure 3:**
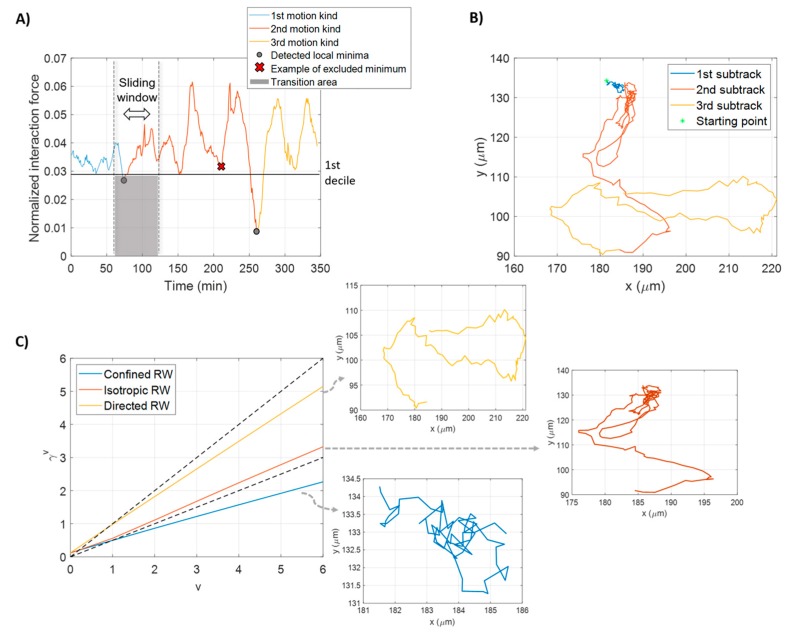
Motility switches detection and motion kind assignment. (**A**) Normalized interaction force exerted by cells in a cluster on a cell belonging to the same cluster. Switches from a mode of motion to another (shown in three diverse colors) match the detected local minima of the force (gray circles), which are located in the transition area (shaded gray area) between the sliding window and the first decile. Minima intercepted by the sliding window, but over the first decile are excluded (red cross). (**B**) The resulting cell trajectory divided in three different-colored sub-tracks corresponding to the three parts obtained by the identification of the local minima in (**A**). The green asterisk denotes the starting point of cell trajectory. (**C**) MSS computation for the three sub-tracks in (**B**) with representation of each sub-track. The slope of the MSS defines the motion kind assignment: 0.3561 for confined random walk (light blue), 0.5432 for isotropic random walk (red) and 0.8368 for random walk with drift (yellow). RW in the legend stands for random walk. Dotted lines with slopes of 1 and 0.5 are also represented as benchmarks.

Local minima of the module of the aforementioned force were identified through a combined use of sliding windowing and first decile computation. Figure 3A shows an example of automatic cell transition mode recognition. First, we eliminated trajectories with a duration smaller than two hours, to keep only the most informative tracks for the analysis. We then imposed the size of the sliding window as a sixth of the trajectory length, a trade-off between obtaining meaningful information from the computation of the local moments of displacement, and avoiding losing information about the interaction force. Too short windows revealed it to be unsuitable to obtain robust local moment signals for the subsequent analysis. On the other hand, longer sliding windows overshadowed the effect of cell interaction.

The first decile was considered as a threshold score in order to detect the lower, and thus the most relevant, local minima. By disposing all of the force values in ascending order, the first decile is the score below which 10% of the values fall. We therefore took as motility switches the solely local minima detected within the sliding window that are below the first decile. They were found in that we called the transition area (represented in gray in the figure). Gray circles represent the two transitions between modes of motion. The red cross locates a minimum identified inside the sliding window, but over the first decile that was excluded, because it fell out of the transition area. After each trajectory was split into a given number of sub-tracks, each tract was automatically assigned to a different kind of motion, as described below.

For the scope of this work, three different kinds of motion were assumed. In the example in Figure 3B, the considered cell track was divided into three tracts identified by different colors. Each sub-track was associated to one kind of three main diffusion modes [18]: *isotropic random walk*, *random walk with drift* and *confined/sub-diffusive random walk*. An isotropic random walk describes cells moving in a succession of random steps, without following a clear-defined direction, a sort of directional trend (drift), which is the hallmark of the directed random walk. A cell mimicking an isotropic or directed random walk does not have constraints during motion, while a cell moving of a confined random walk persists in a restricted area. Motions were discriminated by calculating the Moment Scaling Spectra (MSS) [23,24].

The concept of the MSS is related to that of the *moments of displacements*, including the *Mean Square Displacement* (MSD) [18,19]. Let the two-dimensional position vectors of the $i^{th}$ cell trajectory at time t, $x_{i}\left(t\right)$, with $t=0,1,\ldots L_{i}-1$ with $L_{i}$ the trajectory length. Given a time lag τ, ranging from 1 to $L_{i}/3$ [25], the moment of order v, as a function of the time lag τ, is defined as

(3) $$MoD_{v,i}\left(\tau \right)=\frac{1}{L_{i}-\;\tau }\overset{L_{i}-\;\tau }{\underset{t=1}{\sum }}d{\left(x_{i}\left(t+\tau \right),x_{i}\left(t\right)\right)}^{v},$$

where d denotes the Euclidean distance between two position vectors and v=0,1,…,6 [25].

The second order moment corresponds to the commonly known MSD. Rewriting the power law $MoD_{v,i}\left(\tau \right)$ ∝ $\tau^{\gamma^{v}}$, presented by Sbalzarini et al. [25] as $\log(MoD_{v,i}\left(\tau \right))$ ∝ $\gamma^{v}\log\left(\tau \right)$, the so-called *scaling coefficients* $\gamma^{v}$ were found using a linear least squares fit. 

According to Ferrari et al. [24], the study of all moments of displacements and their related scaling coefficients conveys a robust analysis of the motion analyzed. By plotting the fitted scaling coefficients, $\gamma^{v}$, versus the degrees of moments, v, the MSS is obtained [24,25]. When the MSS plot is a straight-line, it indicates a strong, self-similar process. The slope, computed by a linear least squares’ regression, provides information about the observed motion kind. 

If the MSS slope is around 0.5, the motion is an isotropic random walk (diffusive mode). A directed random walk motion (super-diffusive mode) corresponds to a slope in the region bounded from above by 1 and from below by 0.5. Finally, a confined random walk (sub-diffusive-mode) is observed when the MSS slope is less than 0.5. In Figure 3C, MSSs for each sub-track identified within the single trajectory in the example in Figure 3B were computed and shown. The three tracts were assigned to one motion kind after computing their slopes by fitting. The slope of the MSS in blue is equal to 0.3561, and corresponds to a confined random walk. The MSS in red, with a slope of 0.5432, defines an isotropic random walk. Finally, the slope of MSS in yellow is equal to 0.8368, indicative of a random walk with drift. 

#### 2.4.3. Feature Extraction

A set of commonly used motility features was extracted from each identified sub-track and from the related entire cell trajectory. The mathematical expressions of the computed descriptors may be found in Table 1.

For time-varying descriptors, the corresponding meaningful statistical operators were considered: the average value, some statistical moments of increasing order, i.e., the standard deviation, the skewness, the kurtosis and the average rate of information provided by the distribution of the descriptor values, known as the Shannon entropy [26]. A total of 29 features were estimated for each cell trajectory and the respective identified sub-tracks. Thus, we collected a set of kinematic features computed for the entire trajectories, and another one in which we picked up features for sub-tracks (CMSs). We divided the latter set in three different subsets, each corresponding to a distinct motion kind (isotropic random walk, random walk with drift and confined random walk). All the 29 features were used to build classifiers of our cooperative strategies, but only four of them were exploited for the good teacher sample selection (see sub-paragraphs below).

### 2.5. Machine Learning Architecture 

#### 2.5.1. Data Labeling

We tested the array of CMS device described in the previous paragraph for treatment effect evaluation, i.e., to recognize four diverse experimental conditions (0.0 μM, 0.5−1.0  μM, 5.0 μM and 50.0 μM), corresponding to classes here labeled as 1,2,3 and 4, respectively. Drug concentrations of 0.5 and 1.0 μM were aggregated for data numerosity considerations. 

#### 2.5.2. Good Teacher Sample Selection

According to previous studies [26], we observed that not all of the cells tracks are equally meaningful of drug concentrations effects. For example, isolated cells may not convey the desired information lacking of interacting potential, as well as cells that lie in the core of a cluster that may suffer from limited motility possibility. To serve as a sensor, a cell should be able to receive the drug, to interact with the surrounding, and to change its own motility accordingly. For this reason, we decided to reduce the amount of cell tracks located in the first part of the method to those that have the chance to be CMS. First of all, we identify features directly related to the motility, or that have previously proven to be crucial in a treatment effect study based on cell motility [26]. For this scope, we selected the *average track curvature*, *the distance to cluster center*, *the average and the standard deviation of tangential speed magnitude*. Based on these four descriptors, we eliminated, in a totally unsupervised manner, those data whose feature values, calculated in each single experiment, exceed the 25th or the 75th percentile. The rationale is that kinematics features exceeding those boundaries (curvature and speed) may be related to motionless cells, or cells moving fast in an isolated way. In addition, as elsewhere demonstrated [26], cells that lie in the core of the cluster (small distance to the center) or very far from the center (cells moving from one cluster to another) can be hardly used for a general analysis. 

#### 2.5.3. Cooperative Strategies

For each cell track, the three different modes of motions defined above were recognized. Basically, we implemented a hierarchical cooperative strategy to combine the responses provided by different classification models trained on different modes of motions.

**Figure 4:**
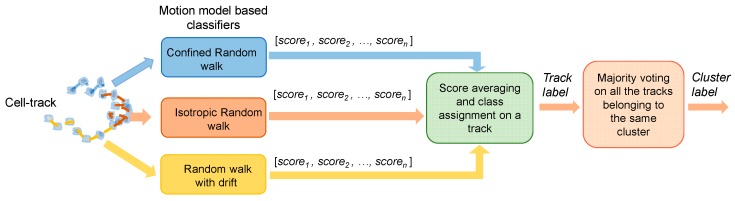
A Schematic block-diagram of the proposed classification platform. From left to right: three classifiers related to the three types of motion are built in order to execute classification on sub-tracks attributed to each kind of these motion models. Each classification model should be able to return a score to belong to a given of n classes (Score_1_, Score_2_, and Score_n_). The score can be averaged class by class, and then the label corresponding to the highest score is assigned to the track (i.e., Track label). The majority voting procedure is then performed at the cell track level, and a label is finally assigned to the entire cluster (i.e., Cluster label).

In a first step, three classification models are independently trained over the sub-tracks (CMSs) assigned to each mode of motions. Let us consider a cell cluster in the test phase. By focusing on one of its cell trajectories, the three models assign to its *n* sub-tracks a score vector (i.e., the selected classification models should be able to return a score to belong to a given class), indicated as *score*_1_, *score*_2_, and *score*_n_ in Figure 4. Secondly, we carried out score averaging at the cell track level, highlighting the cell sensor array potential. The label corresponding to the highest average score is finally assigned to the cell track (*track label* in Figure 4). Finally, the majority voting procedure is applied to the labels of the tracks of the same cluster, thus producing a label at cluster level, i.e., the one with major assignment among the detected CMSs (*cluster label* in Figure 4).

Such a choice is supported by the recently investigated *concerted effort* among cells, emerging in coordinate migration [12,13] and in chemotaxis response [11]. We assume the global behavior in reply to treatment as a concerted effort among cells disposed in the same cluster. Classification model training was performed by a leave-one-cluster-out cross-validation procedure. During simulations, we compared the performances of standard classifiers such as LDA, SVM and QDA. Actually, QDA provided slightly better performance, and hence we selected it for the following analysis. Nevertheless, the rationale for the method was not to focus upon choosing one classifier rather than another, as much as on the optimization of the learning strategy for whichever classification architecture. 

## 3. Results and Discussion

### 3.1. Processed Data

In this work, we tested our platform of analysis on a setting of 14 experiments at diverse experimental conditions, two for the control condition, no drug (0.0 μM), eight for drug concentrations 0.5−1.0 μM, two for 5.0 μM and two for 50.0 μM. With the aim of classifying these four biological conditions, we assigned a label to each of the drug concentrations, i.e., from label = 1 to label = 4 at increasing drug dose. We first applied the automatic clustering, tracking procedure and data refining (see *Methods*), which led to a final result of 794 cell trajectories, 44 for label = 1, 377 for label = 2, 172 for label = 3 and 201 for label = 4, belonging to distinct 119 detected clusters, 12 for label = 1, 63 for label = 2, 17 for label = 3 and 27 for label = 4. Therefore, every single detected cell trajectory was broken up in several sub-tracks by the automatic approach described in *Methods*. By separating sub-tracks according to the relative random walk kind, we collected 819 cell tracks for the isotropic random walk, 819 for a directed random walk, and 829 for the confined random walk, so for a total amount of 2467 sub-tracks. The high number of sub-tracks extracted allowed us to conduct a massive analysis over the cell motility impact of drug administering.

### 3.2. Beyond Univariate Data Analysis

**Figure 5:**
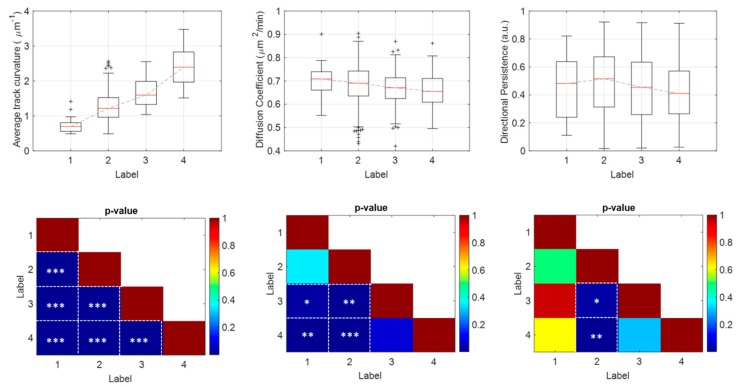
Single-descriptor analysis. In the first row, the box-plots of the average track curvature (left), of the diffusion coefficient (center) and of the directional persistence (right), respectively, extracted from sub-tracks assigned to confined, isotropic and directed random walk, and belonging to the four classes of drug concentration labeled as 1, 2, 3 and 4. In the second row, the triangular matrix of *p*-values for the Student’s t-test test with Bonferroni’s correction corresponding to the three descriptors in the first row. Cells of the matrix with asterisks indicate *p*-values < 0.05. Specifically, * *p*-value < 0.05, ** *p*-value < 0.01, *** *p*-value < 0.001.

The proposed classification procedure is based upon multivariate data analysis to give more robustness to the drug-impact investigation: we combined motility descriptors in a unified array for the three motion models (see *Methods*). Features taken separately, in fact, may not always discriminate efficiently all drug concentrations. As proof of concept, in the first row of Figure 5 the box-plots of the average track curvature for confined random walk (left), of the diffusion coefficient for isotropic random walk (center), and of the directional persistence for random walk with drift (right), at varying drug concentrations, are shown.

The *p*-values for Student’s t-test with Bonferroni’s corrections are represented in the second row of Figure 5. For the average track curvature, the t-test was able to discriminate all concentrations of the drug with *p* < 0.0001 (right). Conversely, the discriminatory ability of the diffusion coefficient fails in discerning the two lower drug concentrations, i.e., classes 1 and 2 (center). Finally, for the directional persistence, a statistically significant difference is exclusively evident by comparing class 2 with class 3, *p* < 0.01, and class 2 with class 4, *p* < 0.001, (right). 

### 3.3. Comparative Approaches

**Figure 6:**
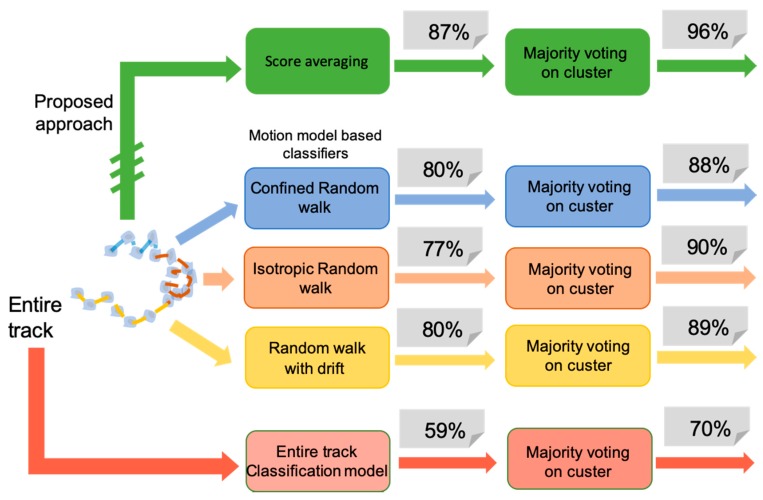
Comparative analysis results. Green boxes at the top indicate the proposed method. The blue, orange and yellow blocks indicate the classifiers constructed on each single motion model, starting from the corresponding sub-tracks of each cell track. Finally, the pink boxes at the bottom indicate the old-paradigm approach in which the entire track is used for the analysis.

To evaluate the advantages of our analysis platform in sensing drug concentration from motility characteristics, we compared the obtained results with those achieved by the alternative approaches indicated in Figure 6. The green blocks identify the proposed method and the related results. Score averaging and class assignment to the single-track reach 87% of the accuracy. Majority voting then produces a strong improvement, and leads to an accuracy value of 96%.

First, we evaluate the performance of individual motion model classifiers by applying the majority voting procedure over the labels assigned, using each motion model separately (intermediate output of the first three rows, blue, orange and yellow blocks). The result obtained by applying the majority voting only to the labels provided by each classifier will be also listed. Results indicate that the three single motion models equally contribute to the final result, but individually does not reach very high performance at track level. When majority voting is used, results improve, but still do not reach the result obtained by their combination. Maximum values of 80% and 90% are obtained, for track and cluster labeling, respectively. The results obtained by the proposed cooperative strategy demonstrates the importance of the “sensor fusion” approach implemented.

To further verify the necessity of a cell motion sensor array strategy, we compared our method with the single-track model approach in which the entire track is extracted for each cell, and its features are used to train the classifier. The cell-track and the cluster level labeling are also indicated in the pink boxes at the bottom. In this approach, a single cell trajectory is considered as a unique sensor, and lacks the potential of constructing an array of sensors within the same cell track. Accuracy results of 59% and 70% support the validity of the novel assumptions. All of the classification models used for the comparison are based on a QDA classifier. 

### 3.4. Discussion

The present study aims at providing a preliminary investigation of the effect of chemotherapy on cancer cell motility during initial time steps after treatment. Indeed, in-vivo cell movements are too complex to be modeled with the scarce nowadays knowledge, and basic information must be gathered before approaching the complexity. In light of this, it seemed wise for us to begin from a simplified model to begin getting basic information, still lacking. The interest in cell motility is due to the fact that cells do not stop moving only because they die, but rather, because the actin movement necessary for locomotion requires specific signaling pathways they may, or may not, depend upon induced DNA damage. Such complexity that manifests much before cell death is not simply the aggregation of multiple cell responses, but rather the synergy of a dynamic cell motility behavior based on heterogeneous motion and the related coordination among different cells. 

To extend the validity of the CMS approach, in future work, the plethora of cell lines will be enlarged at different drug concentrations. In addition, shape descriptors related to morphology changes occurring during cell movement will be joined to motility features for comparative and improved studies. 

## 4. Conclusions

Thanks to the incremented uptake provided by sophisticated camera sensors integrated into time-lapse microscopy (TLM) devices coupled with modern software tools for video and data analysis, cell motion has recently gained increasing interest as a fundamental part of the useful information. We envisaged to consider individual cells themselves as a sort of sensitive element of a sensor. In this view, the camera acts as a transducer, and provides the movement of the cell as an output signal required to retrieve information about the chemical composition of the surrounding environment. According to the fact that cell movement depends on the neighboring environment, in this work we propose to exploit cell movement as the output signal of a “virtual sensor”, with the aim of discovering drug administering impact over cell motility capability. To the best of our knowledge, the dynamics of the phenomena is related to the different motion kinds that a cell assumes during its life cycle. On this basis, we propose and implement here a novel paradigm in which each single cell sub-track can be seen as a sort of sensor, and therefore the entire cell track as a virtual “array of sensors”. Machine learning approaches applied on the descriptors extracted at the sub-track level were demonstrated to be more effective in recognizing the motility effect of the drug. As a proof of concept, we investigated the impact of etoposide (a block replication drug) in PC-3 prostate cancer cells with respect to state-of-the-art methods framed in the standard single-track paradigm. With the support of innovative data refining and cooperative architectures, the proposed platform aggregates the response of individual cell motility-based sensors in a whole, and with an intriguing change of paradigm, presents a very promising tool for further biological investigations and massive drug efficacy studies.

## Figures and Tables

**Table 1:** List of the extracted kinematic descriptors. The index i denotes the $i^{th}$ cell track with coordinates ($x_{i}\left(t_{k}\right),\;y_{i}\left(t_{k}\right)$), for $t_{k}=\;t_{0},\ldots ,t_{f},\;$where $t_{0}$ and $t_{f}$ the starting and the ending time instant of the track. For the track curvature, $x_{i}^{\prime },$ $y_{i}^{\prime }$ and $x_{i}^{\prime \prime },$ $y_{i}^{\prime \prime }$ are the first and second temporal derivatives, respectively, computed by finite difference. For the diffusion coefficient, $y_{0}$ represents the *y*-axis intercept estimated from the Mean Square Displacement sequence.

**Time-Varying Descriptors**	
Tangential speed magnitude [36]	$\;v_{i}\left(t_{k}\right)=\sqrt{{\left(\frac{x_{i}\left(t_{k+1}\right)-x_{i}\left(t_{k}\right)}{\left(t_{k+1}-t_{k}\right)}\right)}^{2}+{\left(\frac{y_{i}\left(t_{k+1}\right)-y_{i}\left(t_{k}\right)}{\left(t_{k+1}-t_{k}\right)}\right)}^{2}}$
Track curvature [26]	$\kappa_{i}\left(t_{k}\right)=\frac{\left|{x_{i}}^{\prime }{y_{i}}^{\prime \prime }-{y_{i}}^{\prime }{x_{i}}^{\prime \prime }\right|}{{\left[{\left({x_{i}}^{\prime }\right)}^{2}+{\left({y_{i}}^{\prime }\right)}^{2}\right]}^{3/2}}$
Angular speed magnitude [26]	$\omega_{i}\left(t_{k}\right)=v_{i}\left(t_{k}\right)\kappa_{i}\left(t_{k}\right)$
Turning angle [36]	$\theta_{i}\left(t_{k}\right)=tan^{-1}\left[\frac{\left(y_{i}\left(t_{k+1}\right)-y_{i}\left(t_{k}\right)\right)}{x_{i}\left(t_{k+1}\right)-x_{i}\left(t_{k}\right)}\right]$
Distance to track center [26]	$r_{C}\left(t_{k}\right)$: the distance of each track point from the corresponding track geometrical center (as the average coordinates in *x* and *y*).
**Constant Descriptors**	
Distance to cluster center [26]	$d_{c}$: the average distance between the geometrical center of the entire cluster (as the average coordinates of all the track centers) and the geometrical center of the track.
Diffusion coefficient [25]	$D_{2}=4^{-1}e^{y_{0}}$
Directional persistence [26]	$p_{i}$: the ratio of the distance between the starting and the ending point of the track and the actual length of the track.
Migration speed [26]	$mv_{i}=\sqrt{{\left(\frac{x_{i}\left(t_{f}\right)-x_{i}\left(t_{0}\right)}{\left(t_{f}-t_{0}\right)}\right)}^{2}+{\left(\frac{y_{i}\left(t_{f}\right)-y_{i}\left(t_{0}\right)}{\left(t_{f}-t_{0}\right)}\right)}^{2}}$
